# Screening of Surfactants for Improved Delivery of Antimicrobials and Poly-Lactic-*co*-Glycolic Acid Particles in Wound Tissue

**DOI:** 10.3390/pharmaceutics13071093

**Published:** 2021-07-17

**Authors:** Fiorenza Rancan, Jana Jurisch, Cemre Günday, Emre Türeli, Ulrike Blume-Peytavi, Annika Vogt, Christoph Schaudinn, Nazende Günday-Türeli

**Affiliations:** 1 Research Center for Hair and Skin Science, Department of Dermatology and Allergy, Charité–Universitätsmedizin Berlin 10117, corporate member of Freie Universität Berlin and Humboldt-Universität zu Berlin, 10117 Berlin, Germany; jana.jurisch@gmx.de (J.J.); ulrike.blume-peytavi@charite.de (U.B.-P.); annika.vogt@charite.de (A.V.); 2 MyBiotech, 66802 Überherrn, Germany; C.Guenday@mybiotech.de (C.G.); e.tuereli@mybiotech.de (E.T.); N.Guenday-Tuereli@mybiotech.de (N.G.-T.); 3 Advanced Light and Electron Microscopy (Zentrum für Biologische Gefahren und Spezielle Pathogene 4), Robert Koch Institute, 13353 Berlin, Germany; schaudinnc@rki.de

**Keywords:** nanoparticles, wound infection, biofilm, *Pseudomonas aeruginosa*, antimicrobial delivery

## Abstract

Topical wound management is often a challenge due to the poor penetration of antimicrobials in wound tissue and across the biofilm matrix where bacteria are embedded. Surfactants have been used for decades to improve the stability of formulations, increase drug solubility, and enhance penetration. In this study, we screened different detergents with respect to their cytotoxicity and their ability to improve the penetration of poly-lactic-*co*-glycolic acid (PLGA) particles in wound tissue. Among the tested surfactants, Kolliphor SLS and Tween 80 increased the penetration of PLGA particles and had a limited cytotoxicity. Then, these surfactants were used to formulate PLGA particles loaded with the poorly water-soluble antibiotic ciprofloxacin. The antimicrobial efficacy of the formulations was tested in a wound infection model based on human ex vivo skin. We found that even though PLGA particles had the same antimicrobial efficiency than the particle-free drug formulation, thanks to their solubilizing and anti-biofilm properties, the surfactants remarkably improved the antimicrobial activity of ciprofloxacin with respect to the drug formulation in water. We conclude that the use of Tween 80 in antimicrobial formulations might be a safe and efficient option to improve the topical antimicrobial management of chronic wound infections.

## 1. Introduction

Surgical or chronic wound infections have a considerable impact on the healthy system [1]. Non-healing wounds such as diabetic foot, pressure, and leg ulcers have an incidence between 1 and 2% [2]. Of two million annual nosocomial infection in the USA, about 60–70% were estimated to be associated with indwelling medical devices [3]. After the introduction of the term biofilm in medicine by Costerton [4], several reports have shown that infections are mainly associated with biofilms i.e., microbial communities encased in an extracellular matrix called extracellular polymeric substance (EPS) [5]. The EPS matrix is a complex mixture of biological polymers such as polysaccharides, proteins, and nucleic acids. The binding of extracellular DNA with the other biopolymers results in a stable 3D structure with strong adhesion properties where bacteria are protected from the external environment. The EPS matrix hides and protects bacteria from the host immune system and is impermeable to various antimicrobial drugs [6,7]. The association of chronic wounds with biofilm infections has been recently confirmed in a meta-analysis where a prevalence of 78.2% of biofilm in chronic wounds was found [8]. In the past, chronic wound infections were considered as a complication of chronic wounds. However, since 2008, the concept of bacterial biofilm being one of the causes of impaired wound healing has been more and more accepted [9,10]. Infected chronic wounds can be treated systemically with antibiotics. However, treatments are often unsuccessful due to insufficient tissue perfusion and, consequently, low drug concentrations at the site of infection. Similarly, topical antimicrobial treatments are often inadequate due to poor drug penetration in deeper layers of the wound or the inability of the drug to cross the bacterial biofilm [11].

Nanoparticles have been widely investigated as transporters for topical drug delivery to wounds [12,13,14]. Being poly-lactic-*co*-glycolic acid (PLGA) a biodegradable polymer, PLGA particles are considered as safe and very promising drug delivery systems [15]. PLGA is an amphipathic polymer that folds into particles with internal hydrophobic regions while having a more hydrophilic surface. Thanks to these internal hydrophobic regions, PLGA particles can encapsulate and transport drugs with low water-solubility [16]. However, particle penetration across biological barriers is limited mainly due to their size and surface charge [17,18,19]. Surfactants have widely been investigated as penetration enhancers in several applications for topical drug delivery to skin, mucosa, and wounds [20,21,22]. Surface-active agents are not only employed as detergents in the management of infected chronic wounds but are also employed in formulations to stabilize drugs and prevent their aggregation and surface adsorption [23].

**Figure A1:**
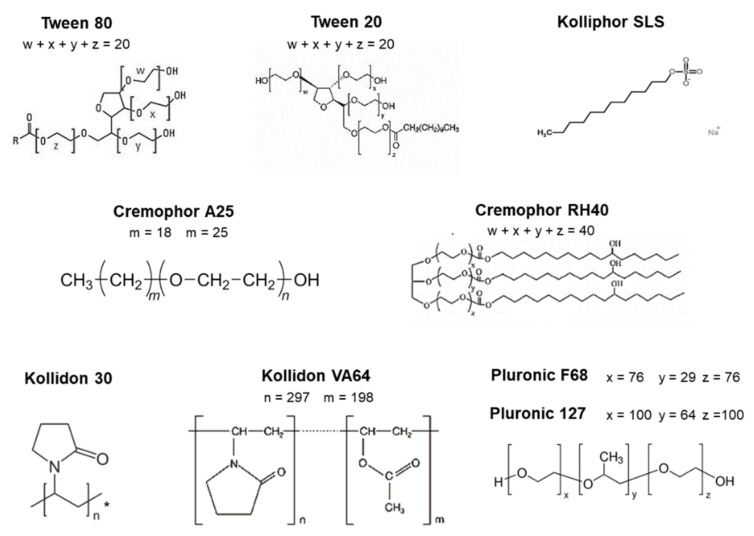
Chemical structures of the investigated surfactants and solubilizers. * Kollidon 30 is a medium molecular povidone with a molecular weight of 44,000–54,000 g/mol.

In this study, we screened different surfactants and solubilizers to be used for the formulation of PLGA particles and improve their penetration in the wound tissue. Non-ionic surfactants and solubilizers were compared to Kolliphor SLS as typical ionic surfactant. The tested penetration enhancers have different hydrophilic–lipophilic balance (HLB) and critical micelle concentration (CMS) (see Table 1), except for Kollidon 30 and Kollidon VA64, which are not amphiphilic and do not for micelles. The chemical structures are given in Figure A1 (Appendix A).

Kolliphor SLS (chemical name: sodium lauryl sulfate) is one on the most used and investigated anionic surfactants. Tween 80 and 20 are polysorbates used in pharmaceutical formulations and food preparation. With regard to antibacterial formulations, both Kolliphor SLS and Tween 80 were shown to increase the stability and as a consequence the antimicrobial efficacy of silver nanoparticles [24]. Poloxamers (such as Pluronic F68 and Pluronic F127) are widely used in wound care, and they have been shown to be safe and to support the wound-healing process [25,26,27,28]. Cremophor A25 is an oil-in-water emulsifier, while Cremophor RH40 is a hydrogenated castor oil used for aqueous cosmetic preparations. Cremophor RH40 has been used in formulations of nanoparticles for the topical delivery of finasteride [29]. Kollidon 30 is polyvinylpyrrolidone, while Kollidon VA64 is a vinylpyrrolidone-based derivative. They are used as binder, film-forming and solubilization agents in a number of pharmaceutical formulations. Polyvinylpyrrolidone is a safe material and is often used in combination with other polymers in the preparation of drug delivery systems for biomedical applications, including wound dressings [30]. 

After examining all formulations with respect to their cytotoxicity and penetration-enhancing properties, the most promising surfactants were used to formulate ciprofloxacin and ciprofloxacin-loaded PLGA particles. The antimicrobial activity of these formulations was tested with a wound model based on ex vivo human skin and inoculated with *Pseudomonas aeruginosa* (PAO-1) bacteria, which was used as an alternative to in vivo animal models [31].

## 2. Materials and Methods

### 2.1. Synthesis and Formulation of PLGA Particles in Different Surfactants

Poly (d,l-lactide-*co*-glycolide) 5050 DLG 4A (Evonik Industries, Essen, Germany) and Lumogen (BASF, Ludwigshafen, Germany) was dissolved in acetone with a concentration of 10 mg/mL and 27.77 µg/mL, respectively. This solution is precipitated as nanoparticles against aqueous surfactant solution with a solvent/non-solvent ratio of 1:2. Surfactants used in the production of nanoparticles were Pluronic F68, Pluronic F127, Cremophor RH40, Cremophor A25, Tween 80, Tween 20, Kollidon VA64, Sodium dodecyl sulfate, and PVP 30. All surfactants were purchased from Sigma-Aldrich Chemie, Steinheim, Germany. Two different concentrations of surfactants, 2.5 mg/mL and 10 mg/mL, were used in the aqueous phase. Nanoparticles were prepared by utilizing MJR technology, where ciprofloxacin (Sigma-Aldrich Chemie, Steinheim, Germany) and PLGA are dissolved in DMSO solution (solvent system) and surfactant (either Koliphor SLS or Tween 80) in water solution (non-solvent system) were delivered to the MJR at 1:5 flow rate ratio at 180° angle by using Smartline S100 pumps (Knauer, Munich, Germany) at room temperature. Purification of the nanoparticles was achieved with Continuous Flow Filtration with hollow fiber membranes (Spectrum Labs, MidiCross, 300 KDa) purchased by Repligen (Ravensburg, Germany).

### 2.2. In Vitro Cytotoxicity Screening (XTT Test)

The concentration-dependent toxicity of the investigated surfactants was measured for the keratinocyte cell line HaCaT and primary dermal fibroblasts. HaCaT cells were purchased from the Deutsches Krebsforschungszentrum (Heidelberg, Germany) and cultured in Gibco 1640 RPMI medium (Thermo Fisher Scientific, Hennigsdorf, Germany) supplemented with 10% FCS (Merck, Darmstadt, Germany), 100 I.E./mL penicillin, and 100 g/mL streptomycin (Sigma-Aldrich, Hamburg, Germany). Primary dermal fibroblasts were isolated from skin explants. Skin was chopped into small pieces, and epidermis was removed with a scalpel. An enzyme cocktail, 3 mg/mL collagenase, 1.5 mg/mL hyaluronidase, and 10 μg/mL DNAse (Sigma-Aldrich Chemie, Steinheim, Germany) was used (1.5 h, 37 °C) to digest connective tissue and isolate cells from the dermis. Cells were collected after filtration (Cell strainer 70, BD Biosciences, Franklin Lakes, NJ, USA) and centrifugation at 216*× g* for 10 min. Then, they were incubated on a 6-well plate overnight. Non-adherent cells were removed, and adherent fibroblasts were cultured in F12 DMEM medium (Lonza, Köln, Germany) supplemented with 10% FCS, penicillin, and streptomycin.

Cells were passaged every two to three days and seeded in 96-well plates for the cytotoxicity tests. After overnight incubation, cells with approximately 50% confluency were exposed with fresh medium mixed with the tested formulations at different concentrations. After 24 h of incubation cell viability was measured using the XTT test (Roche Diagnostic, Mannheim, Germany). The assay is based on the conversion of the yellow tetrazolium salt XTT into an orange formazan dye by the activity of mitochondria dehydrogenase in metabolically active cells. The amount of formazan product was analyzed spectrophotometrically by measuring the absorbance at 492 nm. The reference wavelength was at 650 nm. The optical density (OD) of the blank (XTT in FCS-medium only) was subtracted from the sample OD, and the percentage of cell vitality was determined as follows: (OD sample/OD control) × 100. Bars represent the standard deviations for three independent experiments.

### 2.3. Skin Samples

Excised human skin from the abdominal region was obtained from healthy donors a few hours after plastic surgery (abdominoplasty). The donors had signed an informed consent form. The experiments were conducted following the Declaration of Helsinki guidelines and had been approved by Ethics Committee of the Charité–Universitätsmedizin Berlin (EA1/135/06, 1 July 2015). In total, skin from 12 donors was used, selecting regions without visible lesions or redness. Each experiment was repeated at least three times using skin from different donors. 

### 2.4. Wound Penetration of PLGA Particles Formulated in Different Surfactants 

The wound penetration of labeled PLGA particles formulated in different surfactants was investigated on a wound model based on human ex vivo skin. Skin pieces (1.5 × 1.5 cm) were cut with a scalpel, stretched, and fixed on a Styrofoam block. Then, the wounds were created in the center of the skin using a rotating ball-shaped milling cutter of 6 mm (No. 28725, Proxxon, Föhren, Germany) at 16,000 rpm that was mounted on a dental micro motor handpiece (Marathon N7, TPC Advanced Technology, Inc., Diamond Bar, CA, USA). A volume of 20 µL of PLGA formulations with the surfactants was applied on the top of the wounds using a pipette. Controls received 20 µL of saline. The PLGA concentration was of 5 mg/mL, whereas the surfactants’ concentrations were 2.5 or 10 mg/mL. Samples were placed in an incubator at 37 °C, 5% CO_2_, and 100% humidity. After 20 h, the surface of the wound was rinsed with a cotton swab, the non-treated tissue was removed, and samples were frozen in liquid nitrogen. Skin cryosections of 5 µm thickness were cut with a microtome (2800 Frigocut-N, Reichert-Jung, Heidelberg, Germany). Pictures of at least 20 wound sections (from three different donors) were taken with a confocal laser scan microscope (CLSM, LSM Exciter, Zeiss, Jena, Germany). Lumogen was detected using an excitation wavelength of 550 nm and a detection range of 570–650 nm. Pictures were analyzed with the ImageJ 1.47 software. The mean fluorescence intensity (MFI) of selected areas corresponding to three different penetration depths was calculated and averaged with that of at least 15 other sections. Graphics were created using Excel 2010 (Microsoft Office).

### 2.5. Cellular Uptake of Particles Penetrated in the Wounds

A volume of 20 µL of PLGA particles (10 mg/mL) formulated in 2.5 mg/mL surfactant was applied topically on wounds. Samples were incubated for 20 h (at 37 °C, 5% CO_2_, and 100% humidity) on transwell systems that had inserts with 8 µm pore membranes placed on a 6-well plated filled with 2 mL of RPMI-1640 medium. Then, skin samples were chopped and digested in an enzyme cocktail (3 mg/mL collagenase, 1.5 mg/mL hyaluronidase, and 10 μg/mL DNAse dissolved in PBS with Ca^2+^ and Mg^2+^ and 5% FCS) for 1.5 h at 37 °C. Cells were collected after filtration (Cell strainer 70, and centrifugation at 216× *g* for 10 min. Then, the collected cells were fixed with 4% paraformaldehyde for 10 min and analyzed by flow cytometry (FACS Calibur, Becton Dickinson, Kelberg, Germany). At least 50,000 events were measured. The data were analyzed by the FCSExpress software version 3.1 (De Novo Software, Glendale, CA, USA).

### 2.6. Antimicrobial Activity of Ciprofloxacin Formulations in Ex Vivo Infected Wounds

Wound infection was induced by injecting 5 µL of tryptic soy broth containing 1 × 10^7^ of *Pseudomonas aeruginosa* bacteria (strain PAO1, ATCC 15692) using a 10 µL syringe (26 gauge) (#002000, SGE Analytical Science, Ringwood, Victoria, Australia) from the edge of the wound into the dermis. Wounds inoculated with 5 µL sterile saline (0.9% NaCl) served as controls. After 1 h of incubation in a humidified chamber at 37 °C, ciprofloxacin formulations (20 µL, 0.5 mg/mL) in water or PLGA particles with or without detergent (Tween 80 or Kolliphor^®^ SLS) were applied topically on the top of the wound and incubated for a further 20 h. Each control and sample were run as triplicate in a total of four independent experiments. Using an 8 mm punch biopsy, wound tissue and part of the neighboring tissue were collected, placed in a microcentrifuge tube containing 0.2 mL saline, and homogenized for 3 min with a sterile steel pistil at 150 rpm mounted on a digital overhead stirrer (DSL, VELP Scientifica Srl, Usmate, MB, Italy). To further detach bacteria, samples were sonicated for 10 min in an ultrasonic bath (BactoSonic1, Bandelin, Berlin, Germany) at 40 kHz using 200 W_eff_. The homogenates (100 µL each) were transferred to a 96-well microplate. Seven serial dilutions (1:10) were made by pipetting 20 µL of the concentrated samples and adding 180 µL of saline using a multichannel pipette. Then, 5 µL of each diluted sample was plated on square tryptic soy agar plates. After incubation overnight at 37 °C, spotting areas with 5 to 50 CFU were counted. The CFU per wound was calculated according to the used dilutions and averaged for triplicates. The antimicrobial activity was calculated as a CFU reduction of samples with respect to the CFU of untreated infected wounds. Results are the mean and standard deviation of three independent experiments.

### 2.7. Drug Penetration Kinetics of Cipro in Tween 80 Formulations

For the analysis of drug penetration into the wound, 20 µL of the formulations were applied on the top of the wound and incubated at 37 °C, 5% CO_2_, 100% humidity for 2, 4, 6, 16, and 24 h. Afterwards, the non-penetrated material was removed with a cotton swab, and the tissue surrounding the wound was removed. Then, the wound tissue was chopped in small pieces using a scalpel, and the drug was extracted using 1.5 mL of 0.1 N HCl. Samples were incubated under constant shaking at room temperature for 24 h and then centrifuged for 2 min at 300× *g*. The supernatant (3 × 100 µL) was collected, and fluorescence intensity of ciprofloxacin (excitation wavelength: 275 nm, emission wavelength: 480 nm) was measured with an EnSpire^®^ Multimode plate reader (Perkin Elmer, Akron, OH, USA). A standard curve of ciprofloxacin in 0.1 N HCl (range between 0.5 and 10 µg/mL) was used to calculate the amount of penetrated drug.

### 2.8. Statistics

Means, standard deviations, standard errors, and statistics were calculated and plotted with Excel (Microsoft Corp., Redmond, WA, USA). Statistics was done using one-way ANOVA followed by a Student’s T-test for comparison of two groups of data. 

## 3. Results and Discussion

### 3.1. Evaluation of Surfactant Toxicity toward Keratinocytes and Fibroblasts

**Figure 1:**
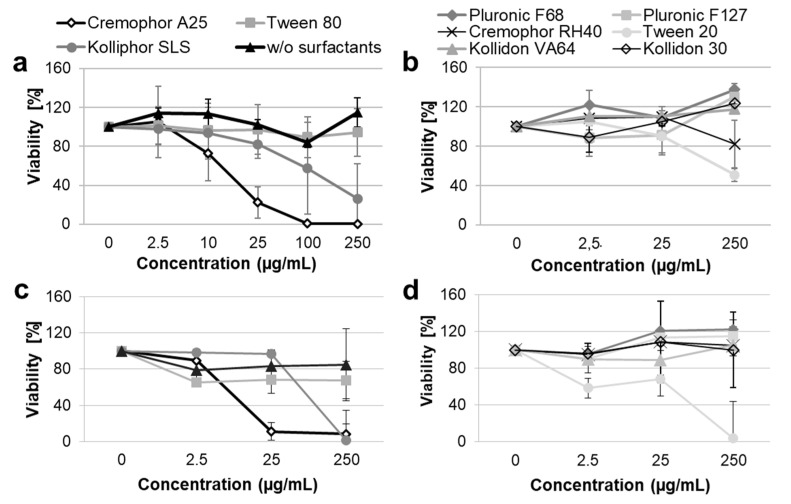
Viability of HaCaT cells (**a**,**b**) and primary dermal fibroblasts (**c**,**d**) after incubation with PLGA particles formulated with different surfactants. Cells were incubated for 24 h with different dilutions of the particle formulations corresponding to different surfactant concentrations. Cell metabolic activity was measured using the XTT colorimetric assay (*n* = 3).

The cytotoxicity of the PLGA formulations in different surfactants was evaluated in vitro using the human keratinocyte cell line HaCaT (Figure 1a,b) and primary human fibroblasts (Figure 1c,d).

A concentration-dependent cytotoxicity was measured for five of the ten tested formulations. The following cytotoxicity ranking was observed: Cremophor A25 > Kolliphor SLS > Tween 20 >> Tween 80 > Cremophor RH40.

Cremophor A25 was the most toxic surfactant, causing approximately 80% dead cells in both keratinocytes and fibroblasts samples already at a concentration of 25 µg/mL. Kolliphor SLS had toxic effects on both cell types at concentrations higher than 25 µg/mL. Tween 20 was more cytotoxic for fibroblasts than for HaCaT cells, with 100% and 50% dead cells respectively after incubation with the highest incubation concentration. The other surfactants had only minimal effects on cell viability. Tween 80 reduced fibroblast viability by approximately 30% at all tested concentrations, while it had no effects on keratinocyte viability. Cremophor RH40 showed marginal cytotoxicity (20% dead cells at the highest tested concentration) toward HaCaT cells only. The PLGA formulations in Pluronic F68, Pluronic F127, Kollidon VA64, Kollidon 30, and in pure water had no effects on cell proliferation and viability at the tested concentrations.

In general, the results found in this study are similar to those reported previously for the same surfactants [32,33,34]. A relationship between toxicity and surfactant physico-chemical properties can be observed. The most toxic surfactants were found to be the ionic surfactant Kolliphor SLS and the non-ionic surfactants with lower HLB of 15–17, whereas non-ionic surfactants with higher HLB (22–29) or hydrophilic polymeric stabilizers such as Kollidon 30 and Kollidon VA64 had low or no toxic effects. This might be explained with the fact that surfactants with saturated (linear) fatty chains with lengths similar to that of cell membrane phospholipids can intercalate the cell membrane and disrupt its integrity better than surfactants with hydrophilic or unsaturated (non-linear) chains [35].

### 3.2. Effects of Different Surfactants on PLGA Particle Penetration in Wound Tissue

**Figure 2:**
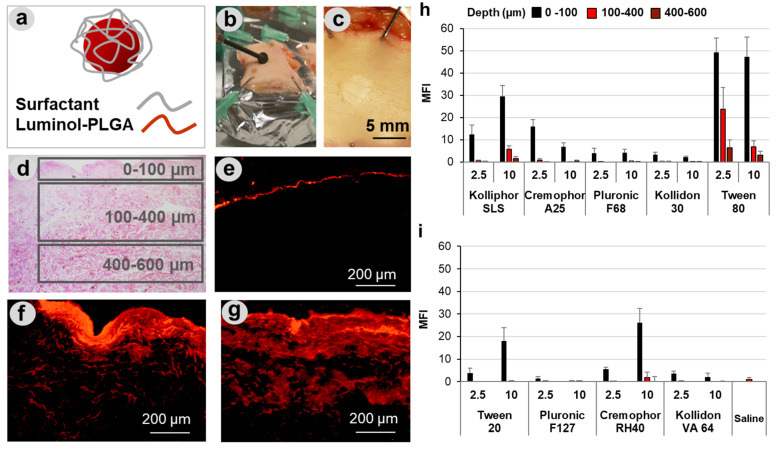
Penetration of PLGA particles in wounds. PLGA particles tagged with the fluorescent dye lumogen and formulated with different types of surfactants (**a**) were applied on superficial wounds created in human skin explants (**b,c**). Cryosections were analyzed considering three areas, corresponding to three penetration depths (**d**). Representative fluorescence images of sections (**e–g**) from samples treated with PLGA particles formulated in water (**e**), Kolliphor® SLS (**f**), and Tween® 80 (**g**) show the enhancement of particle penetration when formulated with surfactants. The diagrams (**h,i**) summarize the average of the mean fluorescence intensities (MFI) in the three penetration areas for particle formulations with two different concentrations of surfactant (2.5 and 10 mg/mL). At least 20 images of skin sections from three different donors were analyzed.

To test the influence of surfactants on particle penetration in wound tissue, lumogen-labeled PLGA particles formulated in different detergents (Figure 2a) were applied on superficial wounds that were mechanically created on ex vivo human skin explants (Figure 2b,c). As shown in the representative image of an H&E-stained section (Figure 2d), the mechanical abrasion treatment resulted in complete removal of the epidermis. Based on section images, three different regions of penetration were considered: superficial (0–100 µm), deep (200–400 µm), and very deep (400–600 µm) (see squares in Figure 2d). As visible in the representative fluorescence microscopy images (Figure 2e–g), the PLGA particles formulated in pure water (Figure 2e) remained at the wound surface, whereas particles formulated with surfactants such as Kolliphor^®^ SLS and Tween^®^ 80 (Figure 2f,g) penetrated to deeper wound regions and reached a maximal depth of approximately 600 µm. The amounts of particles penetrated in the three regions correlated with the fluorescence intensity of the skin sections and were measured with the ImageJ software. The analysis allowed a systematic comparison of the different surfactant formulations (Figure 2 h,i). Five of the nine surfactant formulations clearly enhanced particle penetration in comparison to particle suspensions in water. This positive effect might be due to the interaction of the surfactants with the PLGA particles (i.e., particle stabilization) or with the wound tissue (i.e., tissue hydration and permeabilization). It has been shown that detergents can improve the stability of nanoparticle suspensions. However, this is the case for concentrations below the CMC, whereas at concentrations above the CMC, the stability of particle suspension is usually decreased [36]. Thus, because all surfactants were used at concentrations above the CMC, it is more probable that the observed positive effects are due to the interactions of the surfactants with the tissue components resulting in a perturbation of their ordered structure, hydration of the local environment, and facilitation of particle diffusion. In addition, surfactants may reduce the interaction of particles with skin components, e.g., absorption of proteins, and in this way improve particle diffusion through the connective tissue.

A number of studies have investigated the effects of surfactant on drug and particle penetration across mucus [37,38,39]. It was shown that surfactants can modify the mucus by increasing its hydrophilicity and thus particle diffusion across it [40]. Mucus has a complex composition with electrolytes, lipids, nucleic acids, cell debris, and large glycosylated proteins such as mucin being the main components [41]. Since dermis has a similarly complex composition with collagen and polysaccharides in the extracellular matrix, surfactants may interact with dermis and improve its permeability in a comparable way. Considering the measured average MFI values (Figure 2h,i), which correlate with the number of penetrated particles, the following raking of penetration-enhancing capacity can be done: Tween 80 > Kolliphor SLS > Cremophor RH40 > Cremophor A25 > Tween 20. Nevertheless, only Kolliphor SLS and Tween^®^ 80 formulations resulted in particle penetration into deeper tissue layers between 100–400 and 400–600 µm. The best performance was measured for Tween^®^ 80 that allowed particle penetration even down to 600 µm. Such a deep drug penetration in wound tissue is very important for an efficient topical treatment of infections. In fact, bacteria colonies were found in chronic wounds at depths that correlated to that of the wound bed, and it is believed that also in wounds with no sign of infection, bacteria colonization might have an important impact on wound healing [9,10].

It can be observed that, beside the ionic surfactant Kolliphor SLS, among the non-ionic surfactants with HLB of 13–17, those that mostly enhanced particle penetration (Tween 80 and Cremophor RH40) have a low CMC value (Table 1). These observations can be explained with the fact that surfactants with a higher tendency to form micelles may also better interact with skin components and induce their perturbation, thus promoting PLGA particles penetration in the wound tissue. Comparable results were reported by Zhang et al., who found that PLGA nanoparticle permeation across mucus and the intestinal barrier could be increased by both ionic (SLS) and non-ionic (Tween 80) surfactants with different degrees of penetration enhancement depending on the surfactant physicochemical properties [40].

### 3.3. Effects of Tween^®^ 80 on Particle Intracellular Uptake after Penetration in Wound Tissue

**Figure 3:**
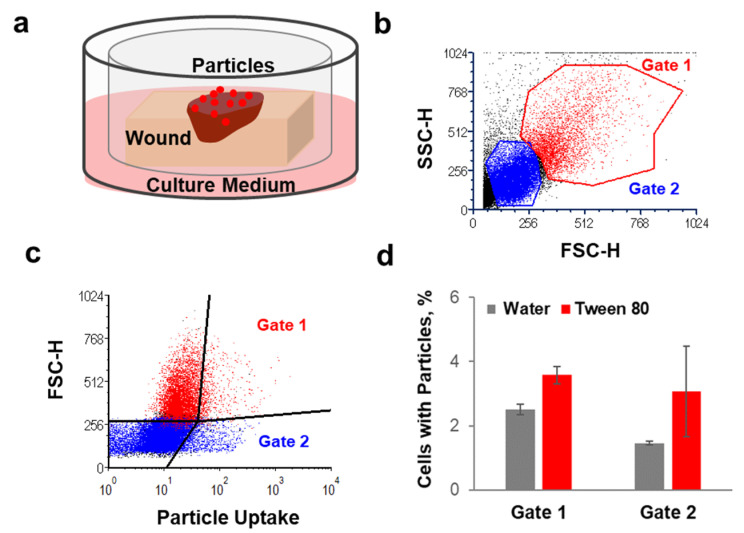
The effects of Tween^®^ 80 formulation on particle uptake by dermis cells. (**a**) Schematic representation of the transwell set-up used for the uptake experiment. (**b**,**c**) Example of the flow cytometry analysis and visualization of the two cell populations considered for the quantification of particle cellular uptake. (**d**) Percentages of cells isolated from dermis that were positive for particle uptake 20 h after topical application of PLGA particle suspensions in water or Tween 80 (*n* = 3).

Tween 80 was found to have the lowest cytotoxicity and the best performance regarding enhancement of PLGA–particle penetration. Therefore, we selected this surfactant formulation to investigate the extent of PLGA particle uptake by skin cells once applied to the wound tissue. For this purpose, we used a short-term organ culture set-up that allowed keeping skin at the air–liquid interface and maintaining cell viability as well as their endocytic capacity [42]. Figure 3a shows a schematic representation of the used transwell set-up. The formulations of lumogen-tagged PLGA particles were applied on the top of the wounds, and after 20 h of incubation at 37 °C, skin cells were extracted from the tissue and analyzed by flow cytometry. Two cell populations were identified according to size (FSC-H) and granularity (SSC-H) (Figure 3b). For the quantification of cellular uptake, cell size (i.e., forward scatter, FSC-H) and lumogen fluorescence (i.e., particle-associated cells) were visualized in dot plots (Figure 3c). Four cell populations were distinguished, and the percentages of cells positive for particle uptake were measured in Gate 1 (high FSC-H and lumogen signal) and in Gate 2 (low FSC-H and high lumogen signal). Gate 1 includes large cells such as fibroblasts and dendritic cells, whereas Gate 2 comprises smaller cells such as T cells and skin resident B cells [43], which have been shown to have phagocyting ability [44].

**Figure A2:**
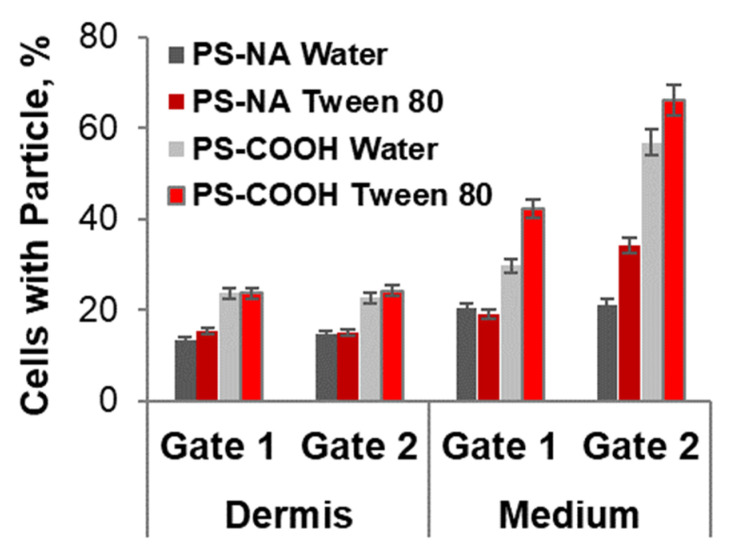
Percentage of cells with associated particles after incubation of ex vivo wounds with 20 mg/mL polystyrene particles (PS, FluoSpheres®, Invitrogen, Carlsbad, CA, USA) functionalized with neutravidin (PS-NA) or carboxyl groups (PS-COOH). Particles had a diameter size of 200 nm and were applied on the surface of wound after a 1:1 dilution of the stock solution with water or Tween 80 (5 mg/mL). The percentages of particle-positive cells were calculated with respect to the total cell number in the analyzed cell population, i.e., cells isolated from wound tissue (dermis) or collected from the lower compartment (medium) after their migration through the 8 µm pores of the insert membrane.

The average of particle-positive cells after topical application of PLGA particles formulated in water or Tween 80 is shown in Figure 3d. Particle cellular uptake was observed for both cell populations. Particle-positive cells after application of the water formulations were 2.5% and 1% in Gate 1 and Gate 2, respectively. The presence of Tween 80 increased particle-positive cells to 3.5% and 3% in Gate 1 and Gate 2, respectively. The positive effect of Tween 80 on particles uptake by skin cells might be due to the improved penetration in the wound tissue and, thus, more interaction with the cells in the dermis. Additionally, the surfactant may have an influence on the formation of protein-corona and aggregation. In fact, the type of protein adhering on the particle surface can determine the extent of intracellular uptake [45] as well as particle aggregation [18]. Interestingly, the number of cells associated with PLGA particles was low in comparison to that found for polystyrene (PS) particles (Figure A2, Appendix A). Such a lower uptake of PLGA particles in comparison to PS was found also in other studies [46,47].

The intracellular uptake was investigated for different reasons. On the one side, intracellular uptake and localization of particles in lysosomes is a desired effect in case of intracellular bacteria such as *S. aureus* [48]. On the other side, extensive particle uptake may be a disadvantage because it reduces the amount of drug delivered to extracellular bacteria or even may have toxic side effects, e.g., in the case of accumulation in immune active phagocyting cells [49]. The fact that the uptake of PLGA particles (with and without surfactants) was very low, especially in comparison to polystyrene nanoparticles (Figure A2), means that the investigated particle formulations are more suitable for the targeting of drugs to extracellular bacteria. Nevertheless, the low rate of intracellular uptake excludes eventual cytotoxic effects associated with activation of immune cells.

### 3.4. Antimicrobial Activity of Ciprofloxacin Formulated with Surfactants

**Figure 4:**
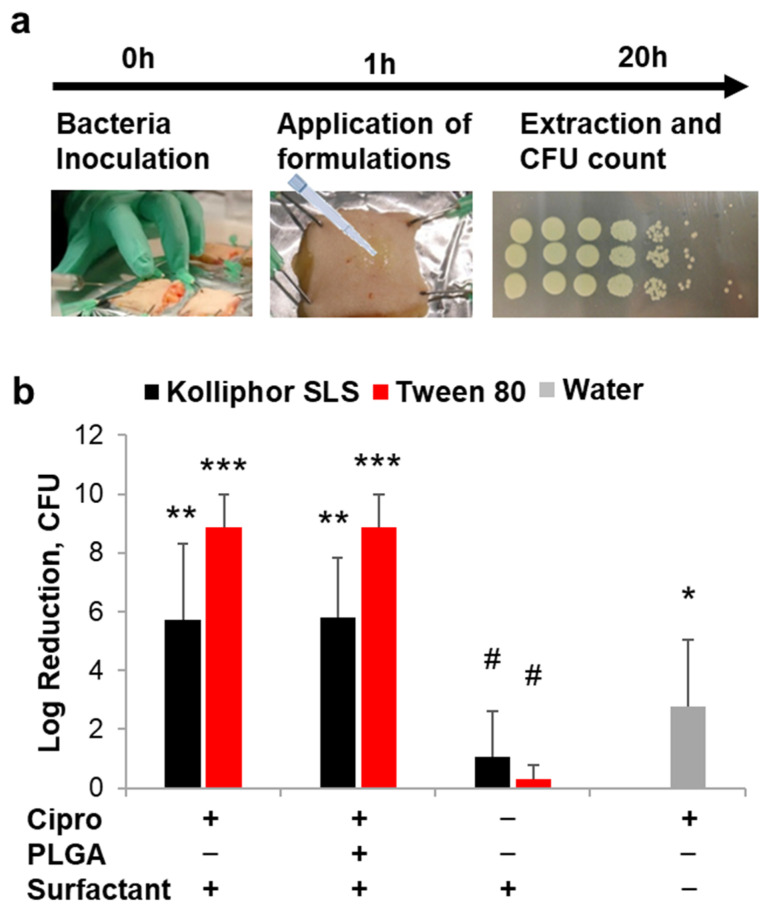
Effects of surfactants on drug delivery and antimicrobial activity. (**a**) Schematic representation of the protocol used to test the antimicrobial efficiency of the tested formulations. (**b**) Antimicrobial activity of ciprofloxacin (applied doses: 10 µg/wound) in different formulations in terms of CFU reduction with respect to untreated infected wounds (*n* = 4). Significance values with respect to untreated controls: *** *p* < 0.001; ** *p* < 0.01; * *p* < 0.05; # *p* > 0.05.

**Figure A3:**
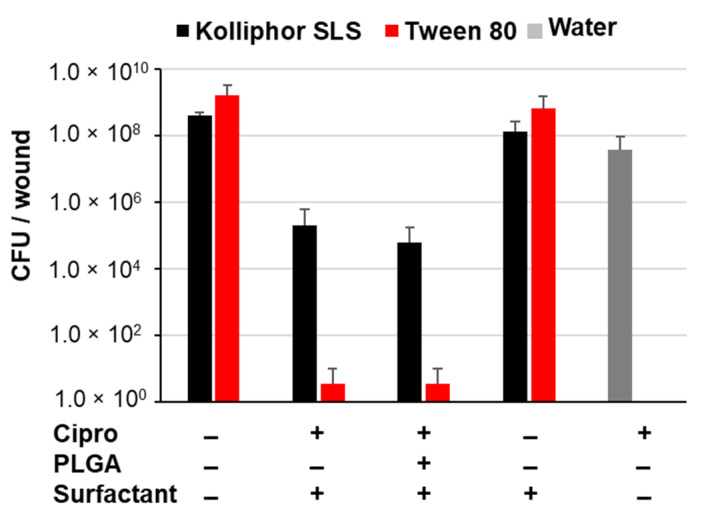
Number of bacteria detected in the wound extracts expressed as colony-forming units (CFU) per wound.

The ex vivo wound model was used to investigate the antimicrobial activity of free ciprofloxacin and ciprofloxacin loaded into PLGA particles formulated in the most promising surfactants Kolliphor SLS and Tween 80. In previous studies, we have demonstrated that the inoculation of *Pseudomonas aeruginosa* in ex vivo wounds results in a rapid growth of bacteria in the tissue and on the surface of the wound with the formation of a biofilm within 20 h of incubation [31,50]. In this study, we applied the antimicrobial formulations 1 h after the inoculation of bacteria in the wound tissue, i.e., before the formation of the bacterial biofilm (Figure 4a). The antimicrobial efficacy of the formulations was evaluated by determining the number of survived bacteria 20 h later using the colony-forming unit (CFU) assay. Formulations of ciprofloxacin and ciprofloxacin loaded on PLGA particles in Kolliphor SLS or Tween 80 were compared to a ciprofloxacin formulation in water. Surfactants alone were used as controls. PLGA particles alone were not tested, being PLGA considered as non-antimicrobial and because of the limited amount of skin biopsies available in each experiment. The antimicrobial activity is plotted as CFU reduction with respect to CFU counted in infected wounds without any treatment (Figure 4b). The CFU counts are given in Figure A3 (Appendix A).

**Figure 5:**
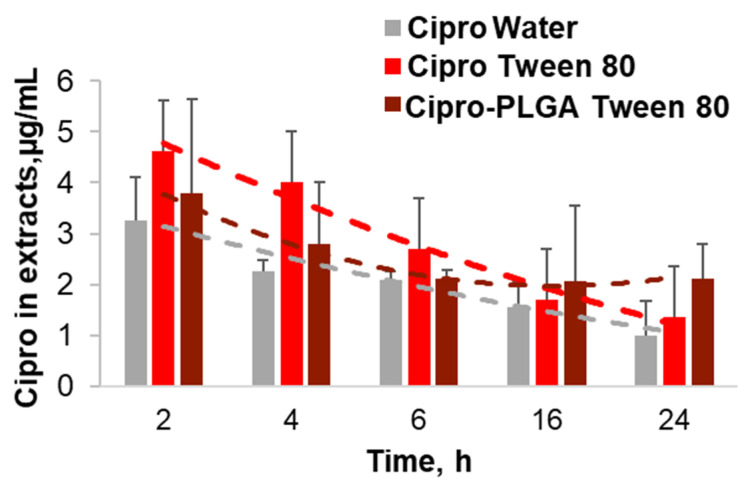
Ciprofloxacin penetration kinetics of Tween® 80 and PLGA-Tween® 80 drug formulations. After incubation of the ex vivo wounds with the different formulations of Cipro in water, Tween 80, and PLGA Tween 80, the drug was extracted and quantified by means of standard curves.

The antimicrobial activity of ciprofloxacin formulated with Tween 80 was superior to that of the antibiotic solution in water (*p* = 0.003 for both free drug and loaded on PLGA particles), whereas low significance was found for Kolliphor SLS formulations (*p* = 0.062 and 0.043 for free drug and loaded on PLGA particles, respectively). Formulations in Tween 80 achieved in average higher Log-reduction values than formulations in Kolliphor SLS (in average 8.8 and 5.8 for Tween 80 and Kolliphor SLS, respectively) with p values of 0.030 and 0.020 when comparing free drug and PLGA particle formulations, respectively. These results might be explained with an increased solubility and penetration of ciprofloxacin in the wound tissue mediated by the surfactants. To verify this hypothesis, the kinetics of drug penetration for formulations in water, Tween 80, and PLGA-Tween 80 was investigated (Figure 5). 

The surfactant formulation achieved higher tissue concentrations than the aqueous drug solution. The highest drug concentrations (≈4.5 µg/wound) were measured for the Tween 80 formulation after short incubation time (2 h and 4 h), whereas the PLGA-Tween 80 formulation achieved lower concentrations in the first hours but resulted in higher concentrations (≈2 µg/wound) with respect to the particle-free formulations at longer incubation times (16 h and 24 h). However, the Tween 80-mediated enhancement of drug concentration in the wound tissue cannot be the only explanation for the excellent antimicrobial activity of the surfactant formulations. A synergistic effect of ciprofloxacin and the surfactant may be possible, even if the surfactants alone exhibited a very low antimicrobial activity. In fact, several reports underline the negative effect of surfactants on biofilm formation [20,51]. In particular, studies using a wound model based on ex vivo porcine skin showed a reduction of biofilm formation using a surfactant-based dressing and evidenced that the inhibition of biofilm formation greatly increased the activity of antimicrobials [52,53]. Thus, we presume that the surfactants Kolliphor SLS and Tween 80 can improve the antimicrobial activity of the low water-soluble drug ciprofloxacin by improving its tissue penetration but also by preventing the formation of biofilm, thus increasing the interaction of the drug with the bacteria cells.

In line with the 3R principles of Replacement, Reduction, and Refinement for humane experimental technique, ex vivo wound infection models have been developed as a tool to reduce in vivo animal studies and linked ethical issues. Ideally, ex vivo models are used to screen potential drugs or devises and hereby to select the most promising candidates for successive in vivo animal or human studies. With respect to in vitro tests with planktonic bacteria or biofilms grown on plastic or glass substrates, experiments on microorganisms grown on ex vivo tissue better reflect the conditions found in wounds. For example, bacteria can adhere to tissue proteins, such as collagen, and need to adapt to the hostile and nutrient-poor environment of skin [31]. On the other hand, ex vivo models have some limitations such as (i) lack of both blood and lymphatic systems, which are crucial for pharmacokinetic studies as well as for tests on the host immune response; (ii) tissue viability, which limits the time span of the experiments [54,55].

Herein, we investigated PLGA particle penetration in wound tissue as a first step to develop drug delivery systems intended to create a depot of antimicrobial drug in the wound tissue. This feature is very important to prevent infection recurrences. However, due to the short time of the experiment with the ex vivo infection model, the antimicrobial effect of a sustained drug release in wound tissue could not be verified. The encapsulation of ciprofloxacin into PLGA particles could slightly change the kinetics of drug delivery (Figure 5), but it did not improve the antibacterial activity within the tested 20 h (Figure 4). Thus, further research with animal models might be necessary to evaluate the possible beneficial effects of PLGA particle depots and sustained drug release in wound tissue.

## 4. Conclusions

In this study, we explored the use of surfactants to improve the penetration of PLGA particles in wound tissue with the aim of developing formulations able to create a depot of antimicrobials in the wound. Based on their low cytotoxicity and penetration-enhancing properties, Kolliphor SLS and Tween 80 were found to be most promising surfactants. The tested surfactants were found to increase the antimicrobial activity of ciprofloxacin formulations, probably thanks to their biofilm-preventing properties, as demonstrated in other studies [56,57]. Surfactants such as poloxamers are used as wound cleansers and have been shown to improve wound healing [20]. Tween 80 is used in skin care products and has been extensively investigated as topical penetration enhancer [58].

Thus, considering that non-healing wounds are very often associated with biofilms, the use of Tween 80 in antimicrobial formulations might be a safe and efficient option to improve the topical management of chronic wound infections.

## Figures and Tables

**Table 1:** Surfactants and solubilizers and their main properties.

Surfactant	Type	HLB ^1^	CMC ^2^
Tween 80	Non-ionic surfactant	15	0.012
Cremophor RH40	Non-ionic surfactant	13–16	0.03
Tween 20	Non-ionic surfactant	16.7	0.06
Cremophor A25	Non-ionic surfactant	15–17	0.08
Pluronic F68	Non-ionic surfactant	29	0.04
Pluronic F127	Non-ionic surfactant	22	0.04
Kolliphor SLS	Ionic surfactant	40	6–8
Kollidon 30	Non-ionic solubilizer	n.a.	n.a.
Kollidon VA64	Non-ionic solubilizer	n.a.	n.a.

^1^ HLB = Hydrophilic/Lipophilic Balance; ^2^ CMC = Critical Micelle Concentration (mM). n.a. = not applicable

## Data Availability

Not applicable.

## Appendix A

*Chemical Structures of Investigated Surfactants*

*Polystyrene Particle Uptake after Topical Application*

*Antimicrobial Activity*
